# Tactile hypo‐responsivity in autism: Examining potential for diagnostic relevance

**DOI:** 10.1002/jcv2.70039

**Published:** 2025-08-13

**Authors:** Girija Kadlaskar, Rebecca McNally Keehn, Riley Rozniarek, Nina Pan Fujii, Brandon Keehn

**Affiliations:** ^1^ Department of Psychiatry and Behavioral Sciences University of California Davis MIND Institute Sacramento California USA; ^2^ Department of Speech Pathology and Audiology University of Nevada Reno Nevada USA; ^3^ Department of Pediatrics Indiana University School of Medicine Indianapolis Indiana USA; ^4^ Speech, Language and Hearing Sciences Purdue University West Lafayette Indiana USA; ^5^ Department of Psychological Sciences Purdue University West Lafayette Indiana USA

**Keywords:** autism, developmental concerns, diagnostic marker, sensory seeking, tactile responsivity

## Abstract

**Background:**

Differences in tactile reactivity are observed in autism and to some extent in children with other developmental concerns. However, it is unknown whether differences in responding to touch may serve as a diagnostic marker of autism in children referred for developmental evaluation. This study examined the utility of a tactile reactivity assessment in differentiating autistic children from those with other neurodevelopmental concerns and the association between tactile responsivity, autism symptomatology, developmental level, and adaptive skills.

**Methods:**

Children (108 autism [mean age 31 months], 43 non‐autism [mean age 29 months]) were administered the tactile portion of the Sensory Processing Assessment along with assessments of autism symptoms, developmental level, and adaptive behavior.

**Results:**

Autistic children showed decreased orienting to both social and non‐social touches compared to children in the non‐autism group. The social touch responsivity had a sensitivity of 0.62 and a specificity of 0.76. The non‐social touch responsivity had a sensitivity of 0.21 and a specificity of 0.93. The sensitivity for overall responsivity was 0.14 and the specificity was 1. For all children, reduced tactile responsivity was associated with greater autism symptomatology, adaptive functioning difficulties and lower developmental levels. Autistic children who engaged in seeking behaviors while playing with distractor toys showed reduced orienting to novel touches.

**Conclusions:**

Autistic children showed reduced orienting to novel touches compared to children with other developmental concerns. Children who did not respond to *both* social and non‐social touches were more likely to be diagnosed with autism. Reduced orienting to tactile stimuli was associated with higher autism symptomatology, and lower verbal/non‐verbal, and adaptive skills in all children. Tendencies of seeking behaviors impacted orienting to touch within the autism group. The findings suggest that tactile reactivity may be a promising indicator of autism diagnosis in young children and that examining this behavior could be a valuable addition to existing standardized protocols.

## Key Points

**What's known:**

- Differences in tactile reactivity are observed in autism and to some extent in children with other developmental concerns.

**What's new:**

- Autistic children show reduced orienting to both social and non‐social touch compared to children with other developmental concerns.
- Children who did not respond to *both* social and non‐social touch are more likely to be in the autism group within the context of a brief tactile assessment.
- Reduced orienting to tactile stimuli is associated with higher autism symptomatology, and reduced verbal, non‐verbal, and adaptive skills in all children.
- Tendencies of sensory seeking may impact orienting to external stimuli within the autism group.

**What's relevant:**

- These findings have implications for clinical practice as they highlight the significance of considering tactile reactivity in autism diagnostic evaluations during early development.

## Graphical abstract

The study reported in this paper examined the utility of a brief tactile reactivity assessment in differentiating autistic children from those with other neurodevelopmental concerns and the association between tactile responsivity, autism symptomatology, developmental level, and adaptive skills. Children who failed to respond to both social and non‐social touches were more likely to be diagnosed with autism. Reduced orienting to tactile stimuli was associated with higher autism symptomatology, and lower verbal/non‐verbal, and adaptive skills in all children.

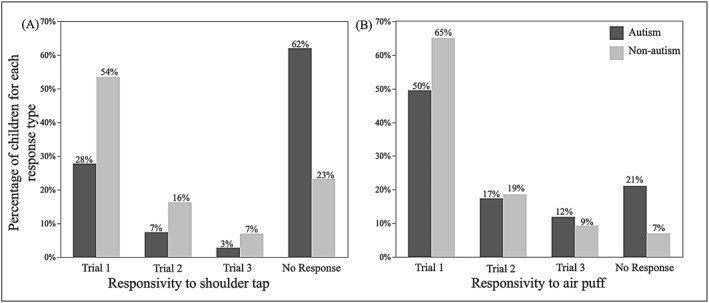

## INTRODUCTION

Autism is a complex neurodevelopmental condition that is characterized by differences in social communication and interaction and the presence of restricted and repetitive behaviors (RRBs) (American Psychiatric Association, 2013). Although symptoms of autism begin to manifest in the first few years of life, autism is typically diagnosed around the age of 4 years in the United States (Maenner et al., 2023). Addressing the gap between symptom onset and diagnosis has the potential to enhance long term outcomes through provision of targeted supports during the critical periods of early development. Timely access to services may contribute to improved adaptive functioning, enhanced social skills and overall greater quality of life for autistic individuals (Fuller & Kaiser, 2020). Past studies have identified several early behaviors such as response to name, use of gestures, vocalization patterns, language delays, attentional orienting, and sensory reactivity that are associated with later autism diagnoses and symptom severity (Zwaigenbaum et al., 2013). Despite these findings, there remains a critical gap in research aimed at utilizing such early autism‐related behaviors to reliably distinguish autistic children from those with other developmental conditions.

One promising area of investigation is the assessment of behavioral orienting responses to novel tactile stimuli in autistic individuals. Touch is one of the earliest sensory modalities to develop and serves as a foundation for other sensory systems, potentially aiding early multisensory development (Bremner et al., 2012; Bremner & Spence, 2017). Touch plays an important role in infants' interactions with their environment and caregivers (Dunbar, 2010). It fosters bonding, secure attachment, and social communication by enhancing positive affect and eliciting responses like smiles and vocalizations (Stack & Muir, 1992). Infants also actively use touch during social interactions and exploration, gaining haptic information about objects and their surroundings. Additionally, touch supports early language development, as caregivers' tactile interactions often accompany language input, facilitating speech perception and comprehension (Seidl et al., 2015). These aspects make tactile responsivity a promising area for identifying early developmental differences, especially in young children.

Research has shown that the majority of individuals with autism exhibit hypo‐reactivity, hyper‐reactivity, and/or seeking behaviors in response to tactile input (Mikkelsen et al., 2018). Hypo‐reactivity is conceptualized as high sensory threshold with passive response whereas hyper‐reactivity is associated with lower sensory threshold and an active response (Dunn, 1997). These differences in responding to tactile stimuli have been extensively reported through first‐person accounts, caregiver reports (Sensory Profile‐2, Sensory Experiences Questionnaire), clinical observations, and behavioral (Sensory Processing Assessment (SPA)) and physiological measures (Baranek et al., 2006; Cascio et al., 2015; Dunn, 2014; Kadlaskar et al., 2021, 2022; Marco et al., 2011b). Moreover, differences in responding to tactile input during early development have been associated with later autism diagnosis and symptomatology. For example, recent prospective studies showed that, 12‐month‐old infants later diagnosed with autism were more likely to not orient to the tactile communication bids initiated by their caregivers during naturalistic play settings (Kadlaskar et al., 2019, 2020). However, it is not known whether a lack of response to tactile stimuli in early development is a reliable indicator of later autism diagnoses.

Although widely reported, differences in tactile responsivity are not unique to autism. In fact, variations in responding to tactile stimuli are observed across a spectrum of developmental conditions, extending beyond autism to include conditions such as global developmental delay (GDD; Barney et al., 2017), Down syndrome (Hennequin et al., 2000), Prader‐Willi syndrome (Priano et al., 2009), Fragile‐X syndrome (Rogers et al., 2003), and other intellectual disabilities (Defrin et al., 2004). While previous research has shown that these tactile reactivity differences are greater in autism (Rogers et al., 2003; Wiggins et al., 2009), other work has suggested that touch responsivity may not differ between autism and DD groups (McCormick et al., 2016). As a result, more research is needed to investigate the utility and specificity of tactile reactivity as an autism diagnostic marker compared to other neurodevelopmental conditions.

The majority of the studies on sensory reactivity, including tactile reactivity, have included individuals with a prior diagnosis of autism or other DDs (Baranek et al., 2007; Boyd et al., 2010; Marco et al., 2011a) or infants who are at elevated likelihood for neurodevelopmental conditions due to family history (Kadlaskar et al., 2019, 2020). These studies offer valuable insights into sensory behaviors linked with neurodevelopmental conditions, also highlighting the trajectories of sensory reactivity before the full phenotypes of these conditions are present. However, there still remains a need for research to explore early markers related to sensory reactivity (including in the tactile modality) that can be reliably used in clinical settings for differential diagnosis of autism. The ability to discriminate between autism and other neurodevelopmental concerns is of crucial importance in both research and clinical practice. Additionally, it is vital to identify reliable behavioral markers that can also be administered by non‐autism specialists in community and primary care settings to determine likelihood of autism or as part of the screening process to facilitate early detection, thereby enhancing outcomes for autistic individuals. This is particularly crucial in contexts where access to specialized autism evaluations may be limited.

The objective of the present study is to examine the utility of a brief tactile reactivity assessment in diagnostic differentiation of autistic children from those with other developmental delays. In order to achieve this goal, we utilized a modified version of the SPA that has been frequently used to measure sensory reactivity differences in autistic children, DD, and TD (Baranek et al., 2013). The modified version of the SPA was specifically chosen for this study because it requires less intensive training than existing diagnostic assessments, is quicker to administer, and relies less on the subjective judgments of examiners. Using the SPA, we examined whether orienting to unexpected delivery of touches reliably differentiates autistic children from those with other developmental concerns within a cohort referred for autism evaluation. Because autistic children may show different orienting responses to social versus non‐social sensory stimuli (Baranek et al., 2013), touch reactivity was examined in both of these domains. Next, because sensory reactivity differences (including for the tactile modality) are linked with social, communication, developmental, and adaptive skills challenges (Foss‐Feig et al., 2012; Kadlaskar et al., 2022; Watson et al., 2011), we also examined the association between orienting to touch and autism symptomatology, developmental level, and adaptive functioning levels across diagnostic groups. We hypothesized that (1) children in the autism group will show greater hypo‐reactivity (i.e., slower or non‐responses) compared to children with other developmental concerns in response to both social and non‐social tactile stimuli, (2) non‐responsivity to social and non‐social touches will differentiate autistic children from those with developmental concerns, and (3) hypo‐reactivity to touch will be associated with greater challenges in social, communication, developmental, and adaptive skills for all children.

Finally, in light of prior findings suggesting the presence of hyper‐reactivity/avoidance and unusual sensory seeking patterns in individuals with neurodevelopmental conditions (Baranek et al., 2007; Kadlaskar et al., 2022), we conducted exploratory analysis to examine whether tendencies of engaging in such behaviors while interacting with distractor toys as part of the SPA (described in detail below) could potentially explain any differences in orienting responses to novel touches. We hypothesized that children exhibiting high sensory thresholds (i.e., showing unusual sensory seeking interests in response to distractor toys) would partially account for differences in children's orienting responses.

## METHODS

### Overview of procedure

Data for the present study were collected as part of a larger study of diagnostic accuracy across a statewide network of community primary care clinicians with specialized training in autism evaluation (McNally Keehn et al., 2023). The larger study was conducted within the framework of the Early Autism Evaluation (EAE) Hub system, a statewide network of community Primary Care Physicians (PCPs) who were trained to perform efficient diagnostic evaluations for young children aged 19–48 months, who were at risk for autism based on developmental screening. The evaluation adhered to a standard clinical protocol and included a developmental history, a focused clinical interview based on the Diagnostic and Statistical Manual of Mental Disorders, Fifth Edition, a physical examination, and the utilization of an observational autism assessment tool (Screening Tool for Autism in Toddlers; Stone et al., 2004). Subsequently, the EAE Hub clinician provided a best‐estimate autism diagnosis when necessary along with a comprehensive report containing clinical recommendations (See McNally Keehn et al., 2023 for more details about the larger study). All children referred by the Hubs to the larger study were re‐evaluated by the study team that included licensed clinical psychologists, clinical research technicians, and postdoctoral researchers as part of a follow‐up visit designed to examine diagnostic accuracy between PCPs and expert autism researchers and clinicians. The study was approved by the Indiana University School of Medicine's Institutional Review Board, and caregivers provided written informed consent.

### Participants

Children were included in the present study if they completed a brief tactile assessment (described below) during their follow‐up visit with the research team. The sample included 19‐ to 48‐month‐old children (*N* = 158; 43 females, 115 males; 112 autism, 46 non‐autism). Participants in the non‐autism group showed global developmental delay, language delay, and/or other emotional, behavioral, or medical concerns (see McNally Keehn et al., 2023 for additional details about the sample included in this study). Autism diagnoses were confirmed by a licensed clinical psychologist with expertise in assessment of autism in young children using the Autism Diagnostic Observation Schedule, Second Edition (ADOS‐2; Lord et al., 2012), Vineland Adaptive Behaviors Scale, Third Edition (VABS‐3; Sparrow et al., 2016), Mullen Scales of Early Learning (MSEL; Mullen, 1995), and a caregiver interview to assess for DSM‐5 (APA, 2000) autism criteria. Assessments were video recorded for analysis (see McNally Keehn et al., 2023 for a detailed description of assessment procedures for the larger study). A total of 28 children (autism = 18, non‐autism = 10) did not have video recordings, but did complete the assessment. Finally, 7 children were excluded from the final sample due to not participating in the tactile assessment (*n* = 5) and experimenter error (*n* = 2). The final sample included 151 children (43 females, 108 males; 108 autism, 43 non‐autism). See Table 1 for participant characteristics.

**TABLE 1:** Participant characteristics.

	Autism (*n* = 108)	Non‐autism (*n* = 43)	*p‐*value
Age (months), *mean* (SD)	31 (7.07)	29 (7.06)	0.13
Gender, *n* (%)			0.02
Female	25 (23%)	18 (42%)	
Male	83 (77%)	25 (58%)	
Race/Ethnicity^a^, *n* (%)			0.04
Asian	1 (0.97%)	0 (0%)	
Black	10 (9.71%)	3 (7.32%)	
Non‐LatinX White	65 (63.11%)	36 (87.80)	
Latine, any race	20 (19.42%)	1 (2.44%)	
More than one race	7 (6.80%)	1 (2.44%)	
Primary caregiver education^b^, *n* (%)			0.49
Less than college	36 (34%)	17 (40%)	
College or higher	71 (66%)	26 (60%)	
Income^c^, *n* (%)			0.35
<$50,000	55 (57%)	20 (49%)	
$50,001–$99,999	26 (27%)	16 (39%)	
≥$100,000	16 (16%)	5 (12%)	
Mullen scales of early learning, *mean* (SD)			
Total DQ	57 (9.63)	76 (16.17)	<0.001
Verbal DQ	44 (19.67)	75 (18.33)	<0.001
Nonverbal DQ	68 (14.54)	85 (14.60)	<0.001
ADOS‐2 CSS^d^, *mean* (SD)	8 (1.65)	3 (1.93)	<0.001

*Note*: Missing data: ^a^Ethinicty: *n* = 5 in autism, *n* = 2 in non‐autism; ^b^Maternal education: *n* = 1 in autism; ^c^Income: *n* = 11 in autism, 2 in non‐autism; ^d^ADOS CSS: *n* = 1 in autism.

Abbreviations: ADOS‐2 CSS, autism diagnostic observation schedule‐2 calibrated severity score; DQ, developmental quotient; SD, standard deviation.

### Measures

#### Mullen scales of early learning

The MSEL is a standardized assessment that measures verbal and nonverbal development in children from birth to 68 months. It consists of five subscales: gross motor, fine motor, visual reception, receptive language, and expressive language (Mullen, 1995). Developmental quotients (DQ) were calculated by dividing the average of age‐equivalent subscale scores (i.e., mental age) by the child's chronological age and multiplying by 100. Verbal DQ (comprised of receptive and expressive subscales), nonverbal DQ (comprised of fine motor and visual reception), and overall DQ (comprised of verbal and nonverbal DQs) were included in the present study.

#### Autism diagnostic observation schedule, second edition

The autism diagnostic observation schedule, second edition (ADOS‐2) is a semi‐structured, standardized assessment of social communication, interaction, play, and RRBs. Consistent with the ADOS‐2 manual, selection of the correct module (e.g., Toddler, Module 1, Module 2) was based on language and developmental level of the child. Calibrated Severity Scores (CSS) ranging from 1 to 10 based on the ADOS‐2 diagnostic algorithm were used as symptom measures, with higher CSS scores reflecting greater symptom severity (Gotham et al., 2009).

#### Vineland adaptive behavior scales, Third Edition

The VABS‐3 is a caregiver interview designed to assess adaptive functioning in four domains: Communication, Daily Living Skills, Socialization, and Motor Skills (Sparrow et al., 2016). The Adaptive Behavior Composite (ABC) score that is derived from the sum of the Communication, Daily Living Skills, and Socialization domain scores was used with lower scores reflecting greater adaptive functioning difficulties.

#### Sensory processing assessment

The SPA is a play‐based observational assessment that provides behavioral presses to elicit sensory hypo‐reactivity, hyper‐reactivity/avoidance, and seeking responses in children ages 6 months to 6 years (Baranek et al., 2013). Hypo‐reactivity is measured by presenting children with a variety of orienting stimuli (e.g., shoulder tap, air puff, sound stick, name call, hand wave, pen light) while they are engaged with a distractor toy (e.g., waterlog, neon slinky, fish) up to a maximum of three trials or until they show a clear behavioral orienting response (i.e., head turn toward the stimuli). Scores of 1, 2, or 3 are assigned during live administration that correspond to the trial during which the child responds; a score of 4 indicates a non‐response. The SPA orienting stimuli are divided across modalities (tactile, auditory, visual) and type (social and non‐social). In order to examine hypo‐reactivity to social and non‐social touch in a controlled environment, a modified version of the SPA was administered in that orienting was observed only in response to the tactile stimuli (shoulder tap, air puff) while the child engaged with a novel distractor toy (e.g., waterlog and neon slinky, respectively). Order of presentation for the shoulder tap and air puff was counterbalanced across children. The SPA demonstrates a high inter‐rater reliability, with ICCs ranging from 0.91 to 0.99 (Baranek, 1999).

##### Measurement of hyper‐reactivity/avoidance and seeking

Video coding of recorded SPA administrations was conducted using the ELAN software (Brugman et al., 2004) to identify any hyper‐reactivity/avoidance and seeking behaviors performed while children engaged with the distractor toys (waterlog, slinky) as part of our exploratory analysis. Consistent with the SPA manual, overall ratings of 0, 1, or 2 of avoidance behaviors (indicative of hyper‐reactivity) were assigned while children played with each distractor toy (waterlog, slinky). Additionally, in order to identify any sensory seeking behaviors in response to distractor toys, frequency and duration of seeking behaviors along with an overall rating of sensory seeking from 0 to 2 (0 = no unusual sensory seeking, 1 = possible or occasional sensory interests, and 2 = definite or frequent sensory interests) were coded (Damiano‐Goodwin et al., 2018; Kirby et al., 2015). See Appendix A for glossary of seeking behaviors. Frequency and duration of avoidance behaviors were not coded, as avoidant behaviors are likely to be single, short duration events (e.g., engaged with the toy, engaged but with caution, completely avoided the toy). Note that, while we use the term *avoidance* in line with prior SPA studies (e.g., Baranek et al., 2007, 2013), we acknowledge that other frameworks have proposed more nuanced approaches to characterizing sensory responsivity (see He et al., 2023; Ward et al., 2019 for broader conceptualizations and alternative operational definitions).

### Statistical analysis

#### Primary analysis

##### Orienting responses

The data representing orienting responses to shoulder tap and air puff exhibited a non‐normal distribution and attempting to transform the data proved ineffective. Consequently, we elected to employ non‐parametric tests to analyze our data. First, Wilcoxon 2‐sample tests were conducted using orienting scores (1–4) to examine behavioral responses to shoulder tap and air puff stimuli between the autism and non‐autism groups. Second, behavioral orienting responses for all children were recoded as dichotomous variables (0 = response to any of the 3 orienting trials, 1 = no response) to conduct sensitivity and specificity analyses aimed at assessing the accuracy of the tactile assessment in identifying autistic children based on the presence or absence of orienting behaviors. Finally, Positive Predictive Value (PPV; probability that children who did not orient meet the criteria for autism) and Negative predictive Value (NPV; probability that children who oriented do not meet the criteria for autism) were also calculated to strengthen our analysis.

##### Associations between SPA measures and clinical characteristics

Spearman's correlations were conducted to examine the association between touch responsivity to shoulder tap and air puff and ADOS‐2 CSS, VABS‐3 ABC scores as well as MSEL‐derived Verbal DQ, Nonverbal DQ and Overall DQ. To increase the robustness of our findings Bonferroni correction was applied to control for multiple comparisons, resulting in a corrected significance level of *p* < 0.01.

#### Exploratory analysis

We conducted exploratory analyses based on video‐coded data to investigate whether engaging in seeking and/or avoidance behaviors during play with the distractor toy could be associated with reduced tactile responsiveness.

##### Avoidance and seeking behaviors

To examine avoidance and seeking behaviors, chi‐square tests were conducted to determine the association of avoidance and seeking scores and diagnostic group membership. Additionally, for seeking behaviors, the percentage of time that children engaged in such behaviors was computed individually for both distractor toys (i.e., waterlog and slinky). This was achieved by dividing the cumulative duration of seeking behaviors for each toy by the overall time spent interacting with that toy, and then multiplying the result by 100. Wilconxon‐2 sample tests were conducted to examine diagnostic group differences in duration of seeking behaviors for waterlog and slinky. Children in both groups who did not have videos (*n* = 18 autism; 10 = non‐autism) were excluded from exploratory analyses based on video‐recorded data.

Next, using available video recordings, we re‐classified children into seeking versus non‐seeking groups and avoiding versus non‐avoiding groups based on their overall seeking and avoidance scores to each of the distractor toys (non‐seeking: seeking score = 0, seeking: seeking score ≥1; non‐avoiding: avoidance score = 0, avoiding: avoidance score ≥1). This was done separately for waterlog and slinky to assess whether orienting responses to the shoulder tap and air puff stimuli were influenced by children's inclination to exhibit seeking or avoidance behaviors while interacting with the distractor toy. The classification into seeking versus non‐seeking and avoiding versus non‐avoiding groups was informed by the need to clearly differentiate between children who exhibited no behaviors indicative of seeking or avoidance versus those who demonstrated at least one instance of such behavior in the present study. Note that, the classification used in this study was based on children's responses within the specific context of this paradigm and does not imply that behaviors indicative of seeking and avoidance are fixed or mutually exclusive across contexts. We acknowledge that sensory behaviors vary depending on context and this classification should be interpreted as an exploratory approach for identifying patterns within the present dataset.

Finally, when we re‐classified children into the seeking versus non‐seeking and avoiding versus non‐avoiding groups, it became evident that only a small number of children engaged in seeking and avoidant behaviors within the non‐autism group (seeking waterlog = 11, seeking slinky = 4; avoidance waterlog = 2, avoidance slinky = 2). Due to the limited sample size in the seeking and avoiding groups in the non‐autism cohort, our exploratory analysis was focused on the autism group.

## RESULTS

### Orienting responses

**FIGURE 1:**
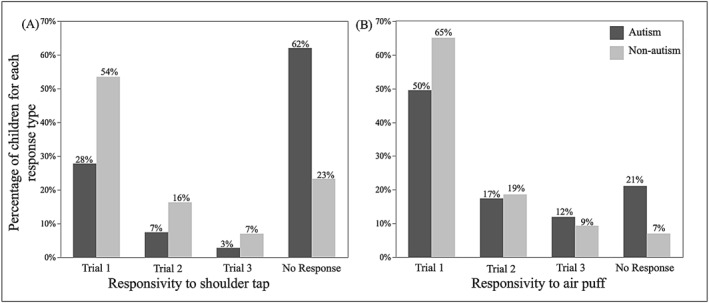
Percentage of children responding to the shoulder tap (A) and air puff (B) stimuli within each group. Scores of 1, 2, and 3 correspond to the trial during which the child responds, 4 indicates a non‐response.

Wilcoxon 2‐sample tests showed that children in the autism group exhibited higher hypo‐reactivity scores, indicating slower orienting or non‐responses, to both the shoulder tap (*Z* = −3.98, *p* < 0.001) and air puff stimuli (*Z* = −2.10, *p* = 0.04) when compared to children in the non‐autism group (Figure 1).

Sensitivity and specificity analyses were separately conducted for orienting responses to the shoulder tap, air puff, and overall orienting to both stimuli. The shoulder tap assessment had a sensitivity of 0.62 (95% CI = 0.52–0.70) and a specificity of 0.76 (95% CI = 0.62–0.86). The PPV of the shoulder tap assessment was 0.87 (95% CI = 0.77–0.92), and the NPV was 0.44 (95% CI = 0.33–0.55). The air puff assessment had a sensitivity of 0.21 (95% CI = 0.14–0.29) and a specificity of 0.93 (95% CI = 0.81–0.97). Air puff assessment PPV was 0.88 (95% CI = 0.71–0.96) and NPV was 0.31 (95% CI = 0.24–0.40). The sensitivity for overall responsivity to touch was 0.14 (95% CI = 0.09–0.22), and the specificity was 1 (95% CI = 0.91–1). The PPV of the overall assessment was 1 (95% CI = 0.80–1) and the NPV was 0.31 (95% CI = 0.24–0.40).

### Associations between SPA measures and clinical characteristics

For all children, higher hypo‐reactivity scores to shoulder tap and air puff (indicative of slower or no responses) were associated with greater autism symptom severity, and lower adaptive functioning and Verbal and Overall DQ (all *p‐*values <0.01; Table 2). Lower Nonverbal DQ was associated with reduced orienting to the shoulder tap. Within the autism group higher hypo‐reactivity scores to shoulder tap were associated with reduced Verbal DQ (*p* < 0.01).

**TABLE 2:** Associations between SPA measures and clinical characteristics.

	ADOS‐2 CSS	VABS‐3 ABC	Total DQ	Verbal DQ	Nonverbal DQ
All participants					
Orienting to shoulder tap (*n* = 151)	0.38**	−0.38**	−0.37**	−0.41**	−0.34**
Orienting to air puff (*n* = 151)	0.21**	−0.20**	−0.21**	−0.24**	−0.20
Autism					
Orienting to shoulder tap (*n* = 108)	0.18	−0.23	−0.22	−0.30**	−0.23
Orienting to air puff (*n* = 108)	0.07	−0.12	−0.16	−0.17	−0.16
Non‐autism					
Orienting to shoulder tap (*n* = 43)	0.32	−0.31	−0.27	−0.26	−0.16
Orienting to air puff (*n* = 43)	0.27	−0.15	−0.11	−0.16	−0.01

*Note*: To validate the robustness of our findings Bonferroni correction was applied to control for multiple comparisons, resulting in a corrected significance level of *p* < 0.01 (0.05/5).

**p < 0.01.

### Exploratory analysis: Understanding possible factors influencing orienting responses to tactile stimuli

#### Avoidance behaviors

There was no significant association between avoidance of the waterlog and diagnostic group, *X* ^*2*^(2, *N* = 124) = 3.51, *p* = 0.17, nor between avoidance of the slinky and diagnostic group, *X* ^*2*^(2, *N* = 124) = 2.38, *p* = 0.30. Within the autism group, membership in the avoiding versus non‐avoiding groups was not associated with orienting to shoulder taps and air puffs (*Z* = 1.15, *p* = 0.25; *Z* = 0.51, *p* = 0.61, respectively).

#### Seeking behaviors

There was no significant association between seeking scores in response to the waterlog and diagnostic group, *X* ^*2*^(2, *N* = 123) = 1.93, *p* = 0.37. However, children in the autism group spent significantly more time seeking the waterlog compared to children in the non‐autism group (*Z* = −2.02, *p* = 0.04). Finally, there was no significant association between seeking scores in response to the slinky and diagnostic group, *X* ^*2*^(2, *N* = 123) = 3.23, *p* = 0.19. Similarly, the two groups did not differ in the amount of time spent seeking the slinky (*Z* = −1.73, *p* = 0.08).

Within the autism group, membership in the seeking group (i.e., engaging in seeking behaviors while playing with the waterlog) was associated with reduced orienting to shoulder taps (*Z* = 2.34, *p* = 0.02). Children who engaged in seeking behaviors while playing with the slinky also showed reduced orienting to air puffs (*Z* = 3.31, *p* < 0.001). Follow up Spearman's correlations showed that spending more time engaged in seeking behaviors in response to the waterlog and slinky was associated with reduced responsiveness to shoulder taps (〉 = 0.28, *p* = 0.01) and air puffs (〉 = 0.31, *p* = 0.002), respectively.

## DISCUSSION

Differences in tactile responsivity are often observed in autistic individuals, and to some extent, in other neurodevelopmental conditions. However, it is unclear whether response to touch may serve as a reliable marker for distinguishing autistic children from those with other developmental concerns. The present study aimed to examine the utility of a brief tactile reactivity assessment in differentiating autistic children from those showing other developmental delays in clinical settings; the assessment involved presenting children with unexpected social and non‐social tactile stimulation while they engaged with a distractor toy. We also conducted exploratory analysis to examine whether sensory thresholds impacted children's orienting responses.

Our results showed that children in the autism group exhibited higher levels of hypo‐reactivity (i.e., reduced orienting) to both social (shoulder tap) and non‐social (air puff) touches compared to those with other developmental concerns. Our sensitivity and specificity analysis showed that within the autism group, 62% of children did not orient to the shoulder tap, compared to 23% in the non‐autism group. Similarly, 21% of children in the autism group did not orient to the air puff in contrast to 7% in the non‐autism group. This suggests that while both diagnostic groups may exhibit some under‐responsivity to novel touches, autistic children were more likely to display under‐reactivity to the tactile stimuli and this difference was more evident in response to social stimuli. These results are consistent with prior studies that have shown that young autistic children show greater sensory challenges compared to children with other developmental concerns (Baranek et al., 2007, 2013; Wiggins et al., 2009) and that hypo‐reactivity to sensory stimuli in both social and non‐social contexts is associated with autism diagnosis (Baranek et al., 2006). Our findings extend these results to the tactile modality within the context of a brief assessment conducted in clinical settings with young children referred for evaluation.

Next, 14% of children in the autism group did not orient to both social and non‐social touches, as compared to no children in the non‐autism group. The specificity of overall touch responsivity for classifying diagnostic outcome was found to be high, with all children in the non‐autism group responding to at least one of the touches. This indicates that not responding to *both* social and non‐social touches within the scope of the present tactile assessment could serve as a distinct indicator of autism during early development when compared with developmental delay, but not all autistic children showed non‐responsiveness to tactile stimuli. These results support prior research indicating that while many individuals with autism show definite sensory challenges, some may show adaptive sensory response patterns across various contexts (Lane et al., 2014). Our findings add to the current literature by highlighting the significance of specifically focusing on behaviors of under‐responsivity during early development in distinguishing autistic children from those without autism.

Our second aim focused on examining the association between orienting to social and non‐social touches and autism symptom severity, adaptive functioning, and developmental level. Results of our correlation analysis suggested that reduced orienting to both the shoulder tap and air puff was associated with greater autism symptom severity and lower adaptive functioning and verbal, non‐verbal and overall developmental level in all children. This association may reflect a cascading effect where challenges in orienting to incoming tactile stimuli (thereby missing out on opportunities to engage with one's communicative partner) may hinder or delay the development of social, verbal, and adaptive skills across both the autism and non‐autism groups during early development (Cascio et al., 2016). Moreover, within the autism group, reduced orienting to shoulder tap in particular, was linked with reduced verbal skills. These results support past studies (Boyd et al., 2010; Foss‐Feig et al., 2012; Williams et al., 2018) and highlight the importance of social response during early development. For example, responding to social input, including to the tactile modality, lays the foundation for social interaction and promotes language learning by allowing children various opportunities to engage in social and language‐rich environments (Kuhl, 2004; Lew‐Williams et al., 2019). Overall, these findings align with a significant body of literature demonstrating that responding to salient social stimuli in one's surroundings during early childhood is crucial for the development of social, verbal, and adaptive skills.

Our exploratory analysis showed that, there were no significant differences in avoidance and seeking scores between the two diagnostic groups as children engaged with both the distractor toys. However, there was a significant difference in the duration of time that children in the autism group spent in seeking the waterlog relative to children in the non‐autism group, suggesting that when interacting with novel sensory toys, children with and without autism may show similar seeking behaviors; however, children in the autism group may engage in these behaviors for a longer duration compared to children in the non‐autism group. It is important to note that the examination of sensory seeking is complex and varies across studies. Some researchers conceptualize sensory seeking in terms of RRBs, while others emphasize more immediate or observable responses to sensory stimuli. In our study, sensory seeking was defined by the children's engagement with distractor toys, which may differ from how sensory seeking is defined in other contexts or studies. Additionally, environmental factors may influence the expression of sensory seeking behaviors, as children may respond differently in novel settings or objects presented during research studies. Future studies could benefit from exploring sensory seeking behaviors in more diverse contexts to better understand how these behaviors manifest across different settings and developmental stages.

Within the autism group, avoidance of distractor toys did not impact orienting responses to both the touches. However, autistic children who exhibited tendencies of sensory seeking while interacting with distractor toys showed reduced orienting to both the shoulder tap and air puff compared to children in the autism group who did not display any seeking behaviors. Additionally, spending more time seeking sensory elements of the distractor toys was associated with reduced orienting to novel tactile inputs. Several factors may contribute to this finding. For example, it is possible that autistic children who engaged in seeking behaviors while interacting with the distractor toy may have been overly focused in exploring the sensory features of the toy, thereby missing out on external tactile stimuli. Alternatively, based on Dunn's model of sensory processing (Dunn, 2007), autistic children who engaged in seeking behaviors could have elevated sensory thresholds, and thus may be more likely to be non‐responsive to subtle tactile input. Future research using physiological measures is needed to validate this hypothesis. Finally, these results could also be attributed to theories of monotropism suggesting that autistic children focus intensely on a narrow range of stimuli, thereby deprioritizing other inputs in their surroundings (Murray et al., 2005). This focused attention may explain increased seeking and reduced orienting behaviors in the autism group. In sum, these results suggest that heterogeneity in processing sensory information in autism (i.e., avoiding or seeking sensory input) and potential differences in attentional patterns may impact the diagnostic utility of tactile orienting responses during evaluations.

The findings of this study have implications for both clinical practice and research. First, incorporating brief tactile assessments into traditional clinical assessment protocols for young children may have the potential to improve diagnostic accuracy in distinguishing between autistic children and those with other developmental concerns. A particular strength of this approach is that a brief tactile orienting task such as the one included in this study requires no specialized clinical training to administer and score, thereby facilitating accurate identification of autism by non‐autism specialists in community and primary care settings. This is particularly crucial where access to assessment services provided by autism‐specialists may be limited. Second, the brevity of such a task may make it particularly suitable for use in diagnostic protocols for young children administered in various clinical and primary care settings. Results also have implications for potential use of this brief assessment in low‐ and middle‐income countries where access to diagnostic services is limited. However, cultural differences in the SPA have not been extensively studied, so further research is needed to explore whether the behaviors being assessed are culturally specific or if the SPA can be effectively adapted across diverse cultural contexts. In sum, these results highlight the importance of integrating tactile reactivity assessments into standard diagnostic protocols, leading to diagnostic accuracy in distinguishing between young autistic children and those with developmental concerns.

Our study is not without limitations. First, the present study utilized the same pairs of distractor toys and orienting items (i.e., waterlog presented with shoulder tap and slinky presented with air puff) as part of the modified SPA. It is possible that varying levels of interest in the waterlog versus slinky may have impacted children's engagement with the toys, thereby influencing their orienting behaviors to shoulder taps and air puffs. Future studies could examine orienting responses to diverse stimuli while the child is engaged with the same toy, aiming to mitigate the impact of children's preferences for certain toys on overall results. Similarly, future research should seek to validate and expand these findings by incorporating a broader range of tactile stimuli. Doing so would enhance the generalizability of findings across different contexts. Second, since the perception and processing of social and non‐social stimuli may be influenced by factors such as control over the situation and the expectations associated with the stimuli, future research should explore how these factors, in addition to the physical characteristics of tactile stimuli (e.g., intensity, duration) contribute to sensory responsivity in autism. Third, while the brief tactile assessment showed high specificity, its sensitivity for overall touch was low. This suggests that although reduced orienting to touch may be a distinguishing feature for some autistic children, many did respond to at least one type of touch presented in our study. Thus, this measure should not be used as a standalone screening method or interpreted as capturing the full range of autism‐related sensory responsivity. Instead, it may be best to incorporate this into broader clinical evaluations to complement existing approaches. Fourth, future studies should aim to recruit more diverse samples to better understand how these findings may apply across different cultural and demographic contexts. Fifth, similar research is needed to compare tactile responsivity as a distinguishing factor between autistic and neurotypical children to examine the validity of this assessment in broader settings. Finally, although our study mainly focused on touch, it is important to acknowledge that autistic individuals often exhibit sensory differences across multiple modalities. Future research should examine multiple modalities to determine whether touch plays a uniquely distinguishing role or whether similar patterns are observed across other sensory modalities.

In conclusion, autistic children showed reduced orienting to both social and non‐social touches compared to children with other developmental disabilities within the context of a brief tactile assessment. Children who did not respond to *both* social and non‐social touches were more likely to be in the autism group. Additionally, reduced orienting to tactile stimuli was associated with higher autism symptomatology, and reduced verbal, non‐verbal, and adaptive skills in all children. Finally, tendencies of seeking behaviors impacted orienting to external tactile cues within the autism group. These findings emphasize the importance of incorporating tactile reactivity into autism diagnostic evaluations, alongside traditional standardized assessments of young children, as well as considering it in intervention planning during early development.

## AUTHOR CONTRIBUTIONS

**Girija Kadlaskar**: Conceptualization; data curation; formal analysis; investigation; methodology; writing—original draft; writing—review and editing. **Rebecca McNally Keehn**: Conceptualization; data curation; funding acquisition; supervision; writing—review and editing. **Riley Rozniarek**: Methodology. **Nina Pan Fujii**: Methodology. **Brandon Keehn**: Conceptualization; data curation; funding acquisition; investigation; methodology; project administration; supervision; writing—review and editing.

## CONFLICT OF INTEREST STATEMENT

The authors declare no conflicts of interest.

## ETHICAL CONSIDERATIONS

The study was approved by the Indiana University School of Medicine's Institutional Review Board (initial approval 8/19/2020; approval/reference number: Validation of Indiana's Early Evaluation Hub System, IU IRB #1806262614), and caregivers provided written informed consent.

## Data Availability

The data that support the findings of this study are available from the corresponding author upon reasonable request.

## APPENDIX A

### Glossary of sensory behaviors

**Table:**

Category		Description
Object‐related	Mouthing/licking	Child brings an object closer to his/her open mouth; may include placing it inside the mouth and licking the object
	Biting	Child bites the object with teeth
	Smelling	Child brings the object closer to nose and smells it
	Sighting	Child shows prolonged visual exploration or inspection of objects from different angles
	Tactile	Child rubs, strokes, or squeezes the object
	Proprioceptive	Child presses or bangs the object against the table
	Spinning	Child spins the object repetitively (more than 3 times) in a row
	Auditory	Child seeks auditory input from the object (e.g., sound of slinky while playing with it)
	Other	Any other object‐related behaviors not recorded above
Body‐related	Flapping	Child repetitively flaps his/her arm or hands while playing with the slinky or the waterlog
	Posturing	Child tenses his/her body or hands while playing with the slinky or the waterlog

## References

[jcv270039-bib-0001] American Psychiatric Association . (2013). Diagnostic and statistical manual of mental disorders (DSM‐5®) (5 ed.). American Psychiatric Association.

[jcv270039-bib-0002] APA . (2000). Diagnostic and statistical manual of mental disorders: DSM‐IV‐TR (4th ed.). American Psychological Association.

[jcv270039-bib-0003] Baranek, G. (1999). Sensory processing assessment for young children (SPA). University of North Carolina at Chapel Hill. Unpublished manuscript.

[jcv270039-bib-0004] Baranek, G. T. , Boyd, B. A. , Poe, M. D. , David, F. J. , & Watson, L. R. (2007). Hyperresponsive sensory patterns in young children with autism, developmental delay, and typical development. American Journal on Mental Retardation, 112(4), 233–245. 10.1352/0895-8017(2007)112[233:hspiyc]2.0.co;2 17559291

[jcv270039-bib-0005] Baranek, G. T. , David, F. , Poe, M. , Stone, W. , & Watson, L. (2006). Sensory Experiences Questionnaire: Discriminating sensory features in young children with autism, developmental delays, and typical development. Journal of Child Psychology and Psychiatry, 47(6), 591–601. 10.1111/j.1469-7610.2005.01546.x 16712636

[jcv270039-bib-0006] Baranek, G. T. , Watson, L. R. , Boyd, B. A. , Poe, M. D. , David, F. J. , & McGuire, L. (2013). Hyporesponsiveness to social and nonsocial sensory stimuli in children with autism, children with developmental delays, and typically developing children. Development and Psychopathology, 25(2), 307–320. 10.1017/s0954579412001071 23627946 PMC3641693

[jcv270039-bib-0007] Barney, C. C. , Tervo, R. , Wilcox, G. L. , & Symons, F. J. (2017). A case‐controlled investigation of tactile reactivity in young children with and without global developmental delay. American Journal on Intellectual and Developmental Disabilities, 122(5), 409–421. 10.1352/1944-7558-122.5.409 28846038

[jcv270039-bib-0008] Boyd, B. A. , Baranek, G. T. , Sideris, J. , Poe, M. D. , Watson, L. R. , Patten, E. , & Miller, H. (2010). Sensory features and repetitive behaviors in children with autism and developmental delays. Autism Research, 3(2), 78–87. 10.1002/aur.124 20437603 PMC3071028

[jcv270039-bib-0009] Bremner, A. , Lewkowicz, D. J. , & Spence, C. (2012). Multisensory development. Oxford University Press.

[jcv270039-bib-0010] Bremner, A. , & Spence, C. (2017). The development of tactile perception. Advances in Child Development and Behavior, 52, 227–268. 10.1016/bs.acdb.2016.12.002 28215286

[jcv270039-bib-0011] Brugman, H. , Russel, A. , & Nijmegen, X. (2004). Annotating multi‐media/multi‐modal resources with ELAN. In LREC.

[jcv270039-bib-0012] Cascio, C. J. , Gu, C. , Schauder, K. B. , Key, A. P. , & Yoder, P. (2015). Somatosensory event‐related potentials and association with tactile behavioral responsiveness patterns in children with ASD. Brain Topography, 28(6), 895–903. 10.1007/s10548-015-0439-1 26016951 PMC4601930

[jcv270039-bib-0013] Cascio, C. J. , Woynaroski, T. , Baranek, G. T. , & Wallace, M. T. (2016). Toward an interdisciplinary approach to understanding sensory function in autism spectrum disorder. Autism Research, 9(9), 920–925. 10.1002/aur.1612 27090878 PMC5564205

[jcv270039-bib-0014] Damiano‐Goodwin, C. R. , Woynaroski, T. G. , Simon, D. M. , Ibañez, L. V. , Murias, M. , Kirby, A. , Newsom, C. R. , Wallace, M. T. , Stone, W. L. , & Cascio, C. J. (2018). Developmental sequelae and neurophysiologic substrates of sensory seeking in infant siblings of children with autism spectrum disorder. Developmental Cognitive Neuroscience, 29, 41–53. 10.1016/j.dcn.2017.08.005 28889988 PMC5812859

[jcv270039-bib-0015] Defrin, R. , Pick, C. G. , Peretz, C. , & Carmeli, E. (2004). A quantitative somatosensory testing of pain threshold in individuals with mental retardation. Pain, 108(1), 58–66. 10.1016/j.pain.2003.12.003 15109508

[jcv270039-bib-0016] Dunbar, R. I. (2010). The social role of touch in humans and primates: Behavioural function and neurobiological mechanisms. Neuroscience & Biobehavioral Reviews, 34(2), 260–268. 10.1016/j.neubiorev.2008.07.001 18662717

[jcv270039-bib-0017] Dunn, W. (1997). The impact of sensory processing abilities on the daily lives of young children and their families: A conceptual model. Infants & Young Children, 9(4), 23–35. 10.1097/00001163-199704000-00005

[jcv270039-bib-0018] Dunn, W. (2007). Supporting children to participate successfully in everyday life by using sensory processing knowledge. Infants and Young Children, 20(2), 84–101. 10.1097/01.iyc.0000264477.05076.5d

[jcv270039-bib-0019] Dunn, W. (2014). Sensory profile‐2. Pearson Publishing.

[jcv270039-bib-0020] Foss‐Feig, J. H. , Heacock, J. L. , & Cascio, C. J. (2012). Tactile responsiveness patterns and their association with core features in autism spectrum disorders. Research in Autism Spectrum Disorders, 6(1), 337–344. 10.1016/j.rasd.2011.06.007 22059092 PMC3207504

[jcv270039-bib-0021] Fuller, E. A. , & Kaiser, A. P. (2020). The effects of early intervention on social communication outcomes for children with autism spectrum disorder: A meta‐analysis. Journal of Autism and Developmental Disorders, 50(5), 1683–1700. 10.1007/s10803-019-03927-z 30805766 PMC7350882

[jcv270039-bib-0022] Gotham, K. , Pickles, A. , & Lord, C. (2009). Standardizing ADOS scores for a measure of severity in autism spectrum disorders. Journal of Autism and Developmental Disorders, 39(5), 693–705. 10.1007/s10803-008-0674-3 19082876 PMC2922918

[jcv270039-bib-0023] He, J. L. , Williams, Z. J. , Harris, A. , Powell, H. , Schaaf, R. , Tavassoli, T. , & Puts, N. A. (2023). A working taxonomy for describing the sensory differences of autism. Molecular Autism, 14(1), 15. 10.1186/s13229-022-00534-1 37041612 PMC10091684

[jcv270039-bib-0024] Hennequin, M. , Morin, C. , & Feine, J. S. (2000). Pain expression and stimulus localisation in individuals with Down's syndrome. Lancet, 356(9245), 1882–1887. 10.1016/s0140-6736(00)03259-1 11130384

[jcv270039-bib-0025] Kadlaskar, G. , Bergmann, S. , McNally Keehn, R. , Seidl, A. , & Keehn, B. (2021). Electrophysiological measures of tactile and auditory processing in children with autism spectrum disorder. Frontiers in Human Neuroscience, 15, 729270. 10.3389/fnhum.2021.729270 35002650 PMC8733620

[jcv270039-bib-0026] Kadlaskar, G. , Mao, P.‐H. , Iosif, A.‐M. , Amaral, D. , Wu Nordahl, C. , & Miller, M. (2022). Patterns of sensory processing in young children with autism: Differences in autism characteristics, adaptive skills, and attentional problems. Autism, 13623613221115951. 10.1177/13623613221115951 PMC9947195 35999699

[jcv270039-bib-0027] Kadlaskar, G. , Seidl, A. , Tager‐Flusberg, H. , Nelson, C. A. , & Keehn, B. (2019). Atypical response to caregiver touch in infants at high risk for autism spectrum disorder. Journal of Autism and Developmental Disorders, 49(3), 2946–2955. 10.1007/s10803-019-04021-0 31016672 PMC6827711

[jcv270039-bib-0028] Kadlaskar, G. , Seidl, A. , Tager‐Flusberg, H. , Nelson, C. A. , & Keehn, B. (2020). Caregiver touch‐speech communication and infant responses in 12‐month‐olds at high risk for autism spectrum disorder. Journal of Autism and Developmental Disorders, 50(3), 1064–1072. 10.1007/s10803-019-04310-8 31754946 PMC12042276

[jcv270039-bib-0029] Kirby, A. V. , Little, L. M. , Schultz, B. , & Baranek, G. T. (2015). Observational characterization of sensory interests, repetitions, and seeking behaviors. American Journal of Occupational Therapy, 69(3), 6903220010p6903220011‐6903220010p6903220019. 10.5014/ajot.2015.015081 PMC5362027 25871592

[jcv270039-bib-0030] Kuhl, P. K. (2004). Early language acquisition: Cracking the speech code. Nature Reviews Neuroscience, 5(11), 831–843. 10.1038/nrn1533 15496861

[jcv270039-bib-0031] Lane, A. E. , Molloy, C. A. , & Bishop, S. L. (2014). Classification of children with A utism S pectrum D isorder by sensory subtype: A case for sensory‐based phenotypes. Autism Research, 7(3), 322–333. 10.1002/aur.1368 24639147

[jcv270039-bib-0032] Lew‐Williams, C. , Ferguson, B. , Abu‐Zhaya, R. , & Seidl, A. (2019). Social touch interacts with infants’ learning of auditory patterns. Developmental Cognitive Neuroscience, 35, 66–74. 10.1016/j.dcn.2017.09.006 29051028 PMC5876072

[jcv270039-bib-0033] Lord, C. , Rutter, M. , DiLavore, P. C. , Risi, S. , Gotham, K. , & Bishop, S. (2012). Autism diagnostic obervation Schedule, second edition. Western Psychological Services.

[jcv270039-bib-0034] Maenner, M. J. , Warren, Z. , Williams, A. R. , Amoakohene, E. , Bakian, A. V. , Bilder, D. A. , Durkin, M. S. , Fitzgerald, R. T. , Furnier, S. M. , Hughes, M. M. , Ladd‐Acosta, C. M. , McArthur, D. , Pas, E. T. , Salinas, A. , Vehorn, A. , Williams, S. , Esler, A. , Grzybowski, A. , Hall‐Lande, J. , …, & Shaw, K. A. (2023). Prevalence and characteristics of autism spectrum disorder among children aged 8 years—Autism and developmental disabilities monitoring network, 11 sites, United States, 2020. MMWR Surveillance Summaries, 72(2), 1–14. 10.15585/mmwr.ss7202a1 PMC10042614 36952288

[jcv270039-bib-0035] Marco, E. J. , Hinkley, L. B. , Hill, S. S. , & Nagarajan, S. S. (2011a). Sensory processing in autism: A review of neurophysiologic findings. Pediatric Research, 69(8), 48–54. 10.1203/pdr.0b013e3182130c54 PMC3086654 21289533

[jcv270039-bib-0036] Marco, E. J. , Hinkley, L. B. , Hill, S. S. , & Nagarajan, S. S. (2011b). Sensory processing in autism: A review of neurophysiologic findings. Pediatric Research, 69(5 Pt 2), 48R–54R. 10.1203/pdr.0b013e3182130c54 PMC3086654 21289533

[jcv270039-bib-0037] McCormick, C. , Hepburn, S. , Young, G. S. , & Rogers, S. J. (2016). Sensory symptoms in children with autism spectrum disorder, other developmental disorders and typical development: A longitudinal study. Autism, 20(5), 572–579. 10.1177/1362361315599755 26395236 PMC4918912

[jcv270039-bib-0038] McNally Keehn, R. , Swigonski, N. , Enneking, B. , Ryan, T. , Monahan, P. , Martin, A. M. , Hamrick, L. , Kadlaskar, G. , Paxton, A. , Ciccarelli, M. , & Keehn, B. (2023). Diagnostic accuracy of primary care clinicians across a statewide system of autism evaluation. Pediatrics, 152(2), e2023061188. 10.1542/peds.2023-061188 37461867 PMC10686684

[jcv270039-bib-0039] Mikkelsen, M. , Wodka, E. L. , Mostofsky, S. H. , & Puts, N. A. (2018). Autism spectrum disorder in the scope of tactile processing. Developmental Cognitive Neuroscience, 29, 140–150. 10.1016/j.dcn.2016.12.005 28089657 PMC5481487

[jcv270039-bib-0040] Mullen, E. M. (1995). Mullen scales of early learning. AGS Circle Pines.

[jcv270039-bib-0041] Murray, D. , Lesser, M. , & Lawson, W. (2005). Attention, monotropism and the diagnostic criteria for autism. Autism, 9(2), 139–156. 10.1177/1362361305051398 15857859

[jcv270039-bib-0042] Priano, L. , Miscio, G. , Grugni, G. , Milano, E. , Baudo, S. , Sellitti, L. , Picconi, R. , & Mauro, A. (2009). On the origin of sensory impairment and altered pain perception in prader‐willi syndrome: A neurophysiological study. European Journal of Pain, 13(8), 829–835. 10.1016/j.ejpain.2008.09.011 18986815

[jcv270039-bib-0043] Rogers, S. J. , Hepburn, S. , & Wehner, E. (2003). Parent reports of sensory symptoms in toddlers with autism and those with other developmental disorders. Journal of Autism and Developmental Disorders, 33(6), 631–642. 10.1023/b:jadd.0000006000.38991.a7 14714932

[jcv270039-bib-0044] Seidl, A. , Tincoff, R. , Baker, C. , & Cristia, A. (2015). Why the body comes first: Effects of experimenter touch on infants' word finding. Developmental Science, 18(1), 155–164. 10.1111/desc.12182 24734895

[jcv270039-bib-0052] Sparrow, S. S. , Cicchetti, D. V. , & Saulnier, C. A. (2016). Vineland adaptive behavior scales (3rd ed.). Pearson.

[jcv270039-bib-0045] Stack, D. M. , & Muir, D. W. (1992). Adult tactile stimulation during face‐to‐face interactions modulates five‐month‐olds' affect and attention. Child Development, 63(6), 1509–1525. 10.2307/1131572 1446566

[jcv270039-bib-0046] Stone, W. L. , Coonrod, E. E. , Turner, L. M. , & Pozdol, S. L. (2004). Psychometric properties of the STAT for early autism screening. Journal of Autism and Developmental Disorders, 34(6), 691–701. 10.1007/s10803-004-5289-8 15679188

[jcv270039-bib-0047] Ward, J. (2019). Individual differences in sensory sensitivity: A synthesizing framework and evidence from normal variation and developmental conditions. Cognitive Neuroscience, 10(3), 139–157. 10.1080/17588928.2018.1557131 30526338

[jcv270039-bib-0048] Watson, L. R. , Patten, E. , Baranek, G. T. , Poe, M. , Boyd, B. A. , Freuler, A. , & Lorenzi, J. (2011). Differential associations between sensory response patterns and language, social, and communication measures in children with autism or other developmental disabilities. Journal of Speech, Language, and Hearing Research, 54(6), 1562–1576. 10.1044/1092-4388(2011/10-0029) PMC3325756 21862675

[jcv270039-bib-0049] Wiggins, L. D. , Robins, D. L. , Bakeman, R. , & Adamson, L. B. (2009). Breif report: Sensory abnormalities as distinguishing symptoms of autism spectrum disorders in young children. Journal of Autism and Developmental Disorders, 39(7), 1087–1091. 10.1007/s10803-009-0711-x 19283461

[jcv270039-bib-0050] Williams, K. , Kirby, A. V. , Watson, L. R. , Sideris, J. , Bulluck, J. , & Baranek, G. T. (2018). Sensory features as predictors of adaptive behaviors: A comparative longitudinal study of children with autism spectrum disorder and other developmental disabilities. Research in Developmental Disabilities, 81, 103–112. 10.1016/j.ridd.2018.07.002 30060977 PMC7473611

[jcv270039-bib-0051] Zwaigenbaum, L. , Bryson, S. , & Garon, N. (2013). Early identification of autism spectrum disorders. Behavioural Brain Research, 251, 133–146. 10.1016/j.bbr.2013.04.004 23588272

